# Cognitive impairment in older people accessing public mental health services across Australia and New Zealand: Implications for clinical practice, workforce development and service provision

**DOI:** 10.1177/00048674241307147

**Published:** 2024-12-26

**Authors:** Roderick McKay, Anne Wand, Gary Cheung

**Affiliations:** 1 School of Medicine, The University of Notre Dame Australia, Sydney, NSW, Australia; 2 School of Clinical Medicine, Discipline of Psychiatry and Mental Health, Faculty of Medicine and Health, University of New South Wales, Sydney, NSW, Australia; 3 Specialty of Psychiatry, Faculty of Medicine and Health, The University of Sydney, Sydney, NSW, Australia; 4 Department of Psychological Medicine, School of Medicine, Faculty of Medical and Health Sciences, The University of Auckland, Auckland, New Zealand

**Keywords:** Cognitive impairment, mental health, dementia, aged care psychiatry, variation in care

## Abstract

Assessment and management of older people with cognitive impairment, especially those associated with psychiatric symptoms; are recognised as core capabilities of old age psychiatrists. Bi-national collections of HoNOS65+/HoNOS reveal that over 40% of older people entering public mental health services across Australia and New Zealand have a clinically significant rating on the HoNOS65+/HoNOS cognitive problem scale, with rates increasing with age, and significant regional variability. The high rates of cognitive impairment in these data reinforce the need for *all* mental health clinicians working with older people to have the capability to assess people with cognitive impairment. Once cognitive impairment is identified, clinicians must be equipped to incorporate the implications into individualised management plans, appropriate referral pathways and community support services. Such skills cannot be the sole responsibility of old age psychiatrists or aged care psychiatry services given the significant number of older people seen by adult mental health services. Regional variability in rates of cognitive impairment raises significant questions regarding variation in service eligibility criteria, equity of access to appropriate mental health care and the availability of a workforce and clinical environments that can meet the needs of older people with cognitive impairment. Finally, psychiatry trainees must learn about working with older people with various degrees of cognitive impairment as part of providing high-quality psychiatric care for an ageing population.

## Introduction

Australia and New Zealand have ageing populations, with limited systematic planning for how mental health services should appropriately respond (Airth and Oelke, 2020). Cognitive impairment is one characteristic of people seeking mental healthcare that increases with age (Reynolds et al., 2022). Assessment and management of older people with cognitive impairment are core capabilities of old age psychiatrists and the services they work in (Reynolds et al., 2022), but less of a focus in general adult mental health services (Cowman et al., 2022).

This capability is most commonly discussed with reference to working with people with ‘behavioural and psychological symptoms of dementia’ (BPSD), with ongoing debate in Australia and New Zealand regarding the role of public mental health services in meeting needs of this cohort (Draper, 2022; Health and Disability Commissioner| Te Toihau Hauora, Hauātanga, 2024). This debate risks obscuring the implications of cognitive impairment occurring for a range of reasons beyond, but including, dementia which may not be recognised on referral or presentation to services. Many older people with cognitive impairment are seen by non-specialist mental health services, particularly when accessing community care or during mental health-related crises (McKay et al., 2024). Clinical and service-level implications of the prevalence of cognitive impairment in older people therefore extend beyond the practice of old age psychiatrists or old age psychiatry services.

Variability in delivery of services for older people with mental health issues has been identified as an important obstacle to improved care (Pagone and Briggs, 2021; Tabatabaei-Jafari et al., 2020). Older people with presenting problems identified primarily as related to mental illness or suicidality are seen as within scope of mental health services, including in the presence of cognitive impairment. However, regional differences emerge in how the presence of dementia or cognitive impairment impacts the application of this principle. People with dementia or cognitive impairment as the primary or presenting issue are usually seen outside of mental health services (Draper, 2022), but people with BPSD in Australia may be seen by national dementia focused services, state geriatric services or aged care psychiatry services (Westera et al.,2022), with significant interstate variation (O’Connor et al., 2018). Guidelines in at least one state, New South Wales, encourage flexibility of response by mental health services based on the range of services available for people with BPSD (NSW Ministry of Health, 2017; NSW Ministry of Health, 2022). How BPSD, which is a collection of behaviours or symptoms in the presence of dementia rather than a diagnosis, is identified as present or absent at referral therefore has potential to significantly impact access to mental health services. In New Zealand, old age psychiatry service funding and resources vary greatly between regions (Copeland et al., 2023), with no dedicated national dementia behaviour service. There has been no published analysis of how variation in service provision in Australia and New Zealand impacts upon the prevalence of cognitive impairment in people seen by mental health services.

The objectives of this viewpoint paper are to highlight the prevalence of cognitive impairment in older people accessing public mental health services across Australia and New Zealand and discuss the implications for service provision and planning. Analysis of publicly available data from the Australian National Outcomes and Casemix Collection (NOCC) (Australian Institute of Health and Welfare, 2023) and New Zealand Programme for the Integration of Mental Health Data (PRIMHD; Te Pou, 2024) collection is presented. This is important reference information required to examine current practice; plan for service improvement; develop informed responses to recommendations of the Royal Commission into Aged Care Quality and Safety in Australia (Pagone and Briggs, 2021) and meet the Chief Ombudsmen’s expectations for aged residential care in New Zealand (Health and Disability; Office of the Ombudsman, 2024). Royal Commission Recommendations 58 and 59 focus on improvements to multidisciplinary and older persons mental health care for people receiving aged care services, with Recommendation 58 including the following challenge to ‘*promulgate standardised eligibility criteria for hospital, community based, and aged care Older Persons Mental Health Services that do not exclude people living with dementia from eligibility for such services*’ (Office of the Interim Inspector-General of Aged Care, 2023). In New Zealand, the Chief Ombudsmen expects residents in aged residential care, which includes people with dementia and BPSD, to enjoy the highest attainable standard of physical and mental health and is supported to access services (Office of the Ombudsman, 2024).

## Methods

### Estimating the prevalence of cognitive impairment in people accessing mental health services from routinely collected outcome data

The national collection of routinely collected measures within public mental health services in Australia, NOCC (Australian Institute of Health and Welfare, 2023), contains ‘gold standard’ data publicly available at www.amhocn.org/nocc-reporting/amhocn-data-portal. PRIMHD is the New Zealand Ministry of Health’s national mental health and addiction information collection of activity and outcomes data. Both NOCC and PRIMHD mandate collection of, and report, the Health of the Nation Outcome Scales for Elderly People (HoNOS65+; Burns et al., 1999), a clinician-rated measure which brings together twelve 5-point scales (scored 0 to 4), including a cognitive scale (Item 4; James et al., 2018). In both Australia and New Zealand, it is possible for the HoNOS (Wing et al., 1998), rather than HoNOS65+, to be used for older people in limited circumstances, primarily outside of old age psychiatry services, although HoNOS data for older people are only available bi-nationally without breakdown beyond ‘65+ years’. Item 4 of the HoNOS65+ has a satisfactory correlation with the Mini-Mental State Examination (Pirkis et al., 2005), with guidance that scores of ⩾2 on Item 4 of the HoNOS or HoNOS65+ scale are ‘clinically significant’ (Burgess et al., 2009). One study suggests this score (⩾2) correlates with screening cut-offs for dementia (Canuto et al., 2007). Descriptors for a score of 2 include ‘frequently disorientated to time’, ‘definite problems learning new information such as names, recollection of recent events’ and ‘difficulty finding way in new or unfamiliar surroundings’ (Burns et al., 1999). For Australia HoNOS65+ data, summary statistics related to a score of ⩾2 on the HoNOS or HoNOS65+ Item 4 were extracted from the two data sets for the financial years 2019–2022. Variation in prevalence of impairment was identified using the variables:

Service type (inpatient or community)
Jurisdiction (state, territory, nation)
Age band (65 and older, with age band sub-analysis)

For comparison, HoNOS Item 4 data were also extracted for younger adults (25 to 64 years) at a national level.

To compare with New Zealand, we requested summary of HoNOS and HoNOS65+ data from Te Pou for the same period, service type and age band as Australia. New Zealand data are reported for the country as a whole. Australian data represent extraction from the national data set rather than compilation of state statistics.

## Results

**Figure 1.**
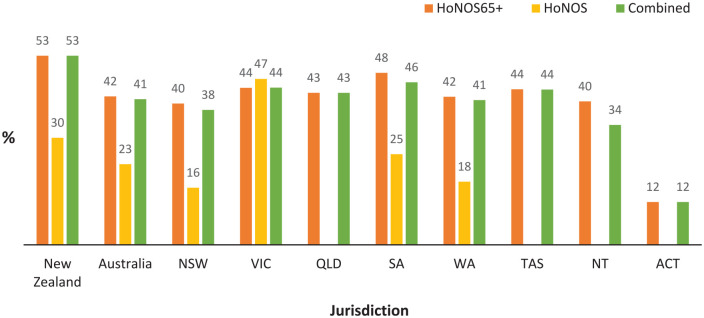
Proportion of consumers aged 65+ with significant^a^ cognitive impairment at admission to community mental health services 2019–2022. Summary statistics are not available where cell size is less than 30. NZ: New Zealand, NSW: New South Wales, VIC: Victoria, OLD: Queensland, SA: South Australia, WA: Western Australia, TAS: Tasmania, NT: North Territory, ACT: Australian Capital Territory. ^a^Significant cognitive impairment defined as score of ⩾2 on Item 4 of HoNOS or HoNOS65+.

**Figure 2.**
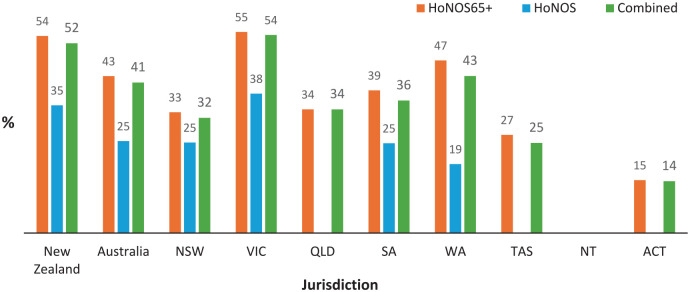
Proportion of consumers aged 65+ years with significant^a^ cognitive impairment at admission to inpatient mental health services in 2019–2022. Summary statistics are not available where cell size is less than 30. NZ: New Zealand, NSW: New South Wales, VIC: Victoria, OLD: Queensland, SA: South Australia, WA: Western Australia, TAS: Tasmania, NT: North Territory, ACT: Australian Capital Territory. ^a^Significant cognitive impairment defined as score of ⩾2 on Item 4 of HoNOS or HoNOS65+.

Figures 1 and 2 show the proportion of older people with identified significant cognitive impairment (i.e. a score of ⩾2 on the HoNOS or HoNOS65+ Item 4) at admission to public mental health community and inpatient services in Australia and New Zealand, respectively. Total numbers of HoNOS/HoNOS65+ collected in each jurisdiction are tabulated at the base of each figure, with Table 1 summarising overall bi-national HoNOS65+ score data. At a national level, over 95% of records are for HoNOS65+ ratings, except New Zealand inpatient ratings where this drops to 79%. Using either HoNOS65+ or combined HoNOS and HoNOS65+ data, over 40% of all people aged 65 years and older accessing Australian public mental health community and inpatient services are identified as having significant cognitive impairment, compared with over 50% in New Zealand. Within Australia, there is significant variation in the identified prevalence of cognitive impairment across states, especially in inpatient settings.

**Table 1.** National HoNOS 65+ Item 4 scores at admission to inpatient and community mental health services 2019–2022.

	New Zealand		Australia	
	Inpatient	Community	Inpatient	Community
*n*	2516	10,120	11,857	37,770
Mean score	1.8	1.7	1.4	1.3
SD	1.3	1.3	1.3	1.2

Prevalence of significant cognitive impairment in younger adult inpatient admissions was 19%/24% (Australia/New Zealand), and 14%/13% at community admission.

**Figure 3.**
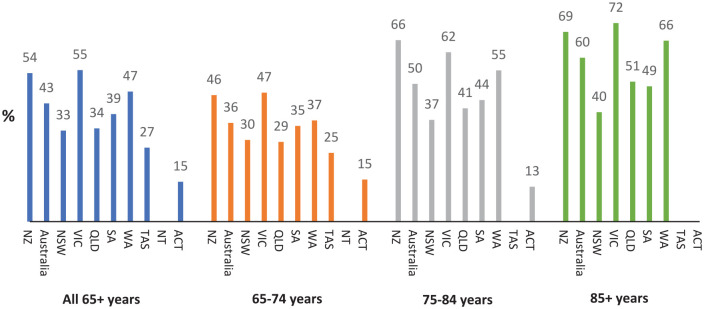
Proportion of consumers aged 65–74, 75–84 and 85+ years with significant^a^ cognitive impairment at admission to inpatient mental health services in 2019-2022. NZ: New Zealand, NSW: New South Wales, VIC: Victoria, OLD: Queensland, SA: South Australia, WA: Western Australia, TAS: Tasmania, NT: North Territory, ACT: Australian Capital Territory. ^a^Significant cognitive impairment defined as score of ⩾2 on Item 4 of HoNOS or HoNOS65+.

**Figure 4.**
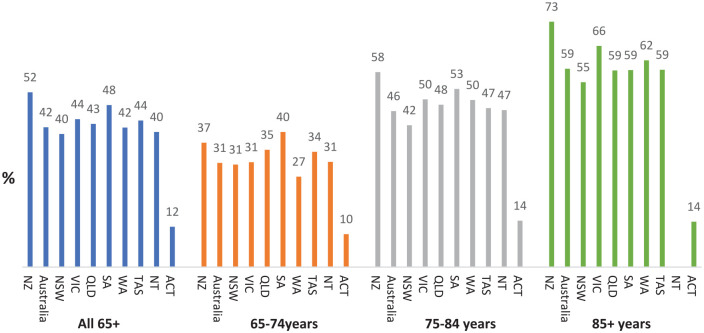
Proportion of consumers aged 65–74, 75–84 and 85+ years with significant^a^ cognitive impairment at admission to community mental health services in 2019-2022. NZ: New Zealand, NSW: New South Wales, VIC: Victoria, OLD: Queensland, SA: South Australia, WA: Western Australia, TAS: Tasmania, NT: North Territory, ACT: Australian Capital Territory. ^a^Significant cognitive impairment defined as score of ⩾2 on Item 4 of HoNOS or HoNOS65+.

Figures 3 and 4 show changes in rates of identified cognitive impairment on the HoNOS65+ by age band across jurisdictions, and associated number of ratings. Rates of people with identified cognitive impairment increase to approximately 60% for people aged 85 or older, but with increasing variation between jurisdictions in both rates, and number of ratings (aligned with number of admissions).

### Data limitations

It is important to first consider the limitations of the data. Collection of routine outcome measures is mandatory at admission to inpatient or community care in Australia and New Zealand, with estimates of completion rates at admission >90% for Australian inpatients (Australian Institute of Health and Welfare, 2024), 80% for significant Australian community episodes (Australian Institute of Health and Welfare, 2024), 80% for New Zealand inpatients and 40% for New Zealand community episodes (personal communication, National Mental Health Informational Development Expert Advisory Panel, 2013; Te Pou, 2024). The NOCC reports portal both enabled this study, and limited data analysis options. We did not include HoNOS/HoNOS65+ scores at discharge, which may affect estimation of the rates of underlying cognitive impairment (assessed by Item 4), as some cognitive impairment may resolve or improve with treatment. Nevertheless, significant cognitive impairment at presentation requires assessment and management. The HoNOS65+ (or HoNOS) score is not diagnostic, nor rated by clinician impression on one item, rather than an objective measure of cognition like the Montreal Cognitive Assessment (Nasreddine et al., 2005), Rowland Universal Dementia Assessment Scale (Storey et al., 2004), or Mini-Mental State Examination (Folstein et al., 1983). There are also minor differences between the HoNOS and HoNOS65+ Item 4 glossaries for cognitive impairment. Cognitive impairment, particularly in the context of dementia, is known to be under-recognised in hospitalised patients and community settings (Cappetta et al., 2022; Ng and Ward, 2019). The data are therefore likely to be an underestimate of cognitive impairment in older people accessing public mental health services. The small volume of HoNOS data means interpretation must be more cautious, but raises the possibility of either lower rates of cognitive impairment in older people accessing adult mental health services or lower rates of recognition. The small sizes of Australian Capital Territory and North Territory data sets also demand caution in interpretation of findings.

## Implications

The findings enhance knowledge about commonalities and variation in the presence of cognitive impairment in older people accessing mental health services. This raises further questions: especially related to why differences occur regionally in rates of cognitive impairment and access in older age.

The key commonality is that significant cognitive impairment is common in people aged 65 years and over who are admitted to public community or inpatient mental health services across Australia and New Zealand, and this prevalence increases with age. However, the prevalence varies significantly across jurisdictions, as does the impact of age on the volume of people with measures (and presumably access to services).

The overall decline in access with age is consistent with national access data in Australia (McKay et al., 2024) suggesting there may also be variable access to specialist mental healthcare in later life. It is essential to consider the implications of both commonalities and variation for practice, policy and workforce development, and whether variation is likely to be ‘warranted’ (to meet individual or population needs or preferences) or ‘unwarranted’ (unable to be explained by such need or values; Duggan et al., 2016; see Box 1). Ideas of what may cause variation require exploration and validation, but action must commence while such investigation occurs.

**Box 1.** Postulated reasons for the prevalence of cognitive impairment across jurisdictions.

Issue location/type	Potentially warranted causes of variation	Potentially unwarranted causes of variation
1. Community	■ Clinical pathways/public information about where to seek help within local service roles ■ Community stigma towards mental health or dementia services	■ Lack of or misinformation regarding service roles ■ Clinician stigma related to mental illness, dementia or ageing
2. Policy	■ Inclusive statements regarding roles of services ■ Funding systems (including activity-based funding) ■ Different mental health acts	■ Lack of service role clarity or integration ■ Funding systems (including activity-based funding) ■ Misinterpretations of mental health act application in people with dementia
3. Broader health system	■ Working relationship between mental health services and geriatric/aged or other services which also provide clinical dementia care	■ Lack of service coordination ■ Varied capacity of residential aged care facilities to work with people with dementia (bed and workforce capacity)
4. Mental health system	■ Clear eligibility criteria ■ Balance of dedicated mental health services for older people and of all-age services	■ Eligibility criteria unclear or unintentionally excludes dementia ■ Intake processes exclude people with dementia ■ Inadequate psychiatry training (e.g. RANZCP Fellowship requirements may be met by two Stage 2 POA-related EPAs rather than a dedicated 6-month rotation) ■ Absence of dedicated old age psychiatry services
5. Broader health system	■ Capacity to assess and work with people with cognitive impairment.	■ Workforce and training issues, e.g. inconsistencies/different threshold to refer or accept patients with mental health concerns and cognitive impairment into mental health services ■ Differing opinions of workforce regarding the role of mental health services

EPAs: entrustable professional activities; POA: psychiatry of old age; RANZCP: Royal Australian and New Zealand College of Psychiatrists.

The high prevalence of significant cognitive impairment highlights why the assessment and management of older people with this comorbidity should be a core element of the mental healthcare of older people, regardless of the mental health service they present to. In clinical practice, cognitive and functional assessment must be standard components of assessment of all older people seen by mental health services. Similarly, all clinicians should have the ability to interpret the diagnostic and management implications of cognitive findings and to sensitively and effectively communicate them to consumers and their families (Foxe et al., 2024; Low et al., 2019). While a proportion of people with cognitive impairment will have dementia, a proportion will have reversible impairment related to mental illness, delirium or other treatable conditions. (Reynolds et al., 2022).

The levels of cognitive impairment found highlight risks associated with assumptions, and clinical decisions, made in response to statements about mental health services having a limited role working with people with dementia (Draper, 2022). In part, the statements and assumptions appear based on low rates of dementia as a *primary* coded diagnosis (Australian Institute of Health and Welfare, 2023) within mental health services; service role descriptions made without consideration of dementia or older people (Queensland Health, 2020), and omissions in Australian national mental health (Department of Health, 2017) and dementia policy (Australian Government Department of Health and Aged Care, 2024) regarding the role of mental health services with people with dementia or cognitive impairment. The high prevalence of cognitive impairment shows that people with cognitive impairment should be recognised as part of core business for mental health services, rather than a reason for potential exclusion from care. The regional variation suggests the need for mental health intake services to be inclusive of people with cognitive impairment presenting with psychiatric symptoms using a ‘no wrong door approach’ (Mental Health Network, 2018). Apart from ethical principles of not excluding people with cognitive impairment from mental health care, there is evidence of better outcomes from such an approach. Mislabelling of cognitive impairment as dementia at point of referral is not uncommon, and reversible causes of cognitive impairment are unlikely to be identified and treated without expert assessment. Contact with ambulatory mental health services has been associated with reduced all-cause mortality in people with self-harm and dementia (Walker et al., 2024).

Internationally, there has been recognition that cognitive assessment is inconsistently conducted by mental health clinicians, including psychiatrists, in adult (Belgaied et al., 2014) and aged care (Newton et al., 2024) psychiatry services. Clinicians need training to identify and work with people with cognitive impairment, including dementia, and to become familiar with referral pathways for further assessment and management of cognitive disorders, as well as services available to support function and carers of older people with mental health challenges and cognitive impairment (Davies, 2023; Reynolds et al., 2022). This is particularly important in triage and acute assessment services, where there is potential for access to be denied based on diagnosis rather than mental health need. From a policy perspective, these findings highlight the artificiality of the ‘dementia/mental illness’ divide present in much mental health and dementia policy at Australian and state levels (Draper, 2022).

The increasing proportion of cognitive impairment with age is unsurprising but emphasises the need to plan for increasing numbers of people with cognitive impairment who will seek mental healthcare. However, the variation between settings and jurisdictions raises serious questions about how policy (and/or training) is impacting on access to appropriate holistic mental healthcare. A degree of variation may be appropriate if it represents adaptation for local variations in service delivery between mental health and geriatric services. However, the degree of variation identified here seems unlikely to be accounted for by such differences, and more likely represents variable access to any care. The interstate variation also makes coordinated care with nationally organised dementia services in Australia problematic (Westera et al., 2022). These challenges may be evidenced in the increasing population rates of older people with mental health disorders presenting to emergency departments (McKay et al., 2024).

The variation of mental health service detection of cognitive impairment in older people between states within Australia, and between Australia and New Zealand, also raises questions regarding the ability of mental health services to consistently provide the training opportunities essential to developing the skills of future psychiatrists to work with older people with cognitive impairment. Currently, psychiatry trainees do not have a mandatory psychiatry of old age rotation, and can meet Royal Australian and New Zealand College of Psychiatrists (RANZCP) Fellowship requirement by completing two psychiatry of old age-specific entrustable professional activities (EPAs) with any psychiatrist (not specifically trained/accredited in psychiatry of old age). Without consideration for how experience and competence in psychiatry of age can be achieved, the planned rationalisation of EPAs in the RANZCP Fellowship programme (RANZCP, 2023) may further erode training opportunities.

Systemic and process changes are needed within public mental health services to *routinely* incorporate the assessment of cognition and function into mental health assessments. At a minimum, this requires identification of appropriate brief cognitive assessment tools (Cowman et al., 2022; Newton et al., 2024), and service protocols expecting their use (Newton et al., 2024). Senior staff need to model how to sensitively conduct these, and more detailed, assessments and incorporate findings into the formulation and management plan, including communicating findings to the consumer and their family. This requires support from improved access to multidisciplinary expertise (McKay et al., in press; Reynolds et al., 2022), able to match cognition and function to individualised strategies and services. People with more complexity require facilitated access to aged care services and cognition clinics (Alty et al., 2023), while mental health teams continue to manage comorbid mental illness.

## Conclusion

Cognitive impairment is common in people aged 65 years and older entering mental healthcare in both Australia and New Zealand. The variation in identified cognitive impairment is great enough that it must impact upon both care and workforce training.

The key question that must be asked is what variation is warranted? Warranted variation results in better experience and outcomes, whereas unwarranted variation risks poorer experience and outcomes, at significant cost. The challenge for clinicians, researchers, policymakers, and service managers is to differentiate between these and identify actions that reduce unwarranted variation. One element of a response must be to work towards *all* mental health clinicians seeing older people in any clinical setting being able to assess cognition without discrimination arising from such detection, and to develop confidence to work with increasing numbers of this population. This must be supported by actions to develop future specialist old age psychiatry workforces with enhanced expertise in working with people with cognitive impairment, and not be subsumed within broader ‘ageless’ services (RANZCP, 2019). While national actions are important, close collaboration and communication between relevant services *at a local level* (RANZCP, 2022) will remain central to preventing vulnerable older people falling between service gaps.
